# Understanding symptoms in the lives of adult patients with acute or chronic illness: a phenomenological study of patient experiences

**DOI:** 10.1080/17482631.2025.2534871

**Published:** 2025-07-30

**Authors:** Malene Missel, Linda Kahr Andersen, Camilla Corvinius, Maria Camilla Mathiasen, Pernille Orloff Donsel, Mai Nanna Schønau, Helle Pappot, Nanna Witting, Melissa Culligan, Giulia Locatelli, Mary Jarden, Karin Piil

**Affiliations:** a Department of Cardiothoracic surgery, Copenhagen University Hospital – Rigshospitalet, Copenhagen, Denmark; b Department of Clinical Medicine, University of Copenhagen, København, Denmark; c Copenhagen Neuromuscular Center, Department of Neurology, Copenhagen University Hospital – Rigshospitalet, Copenhagen, Denmark; d Department of Oncology, Copenhagen University Hospital – Rigshospitalet, Copenhagen, Denmark; e Thoracic Surgery Research, Department of Thoracic Medicine and Surgery, Temple University Hospital Philadelphia, Philadelphia, PA, USA; f Department of Medicine and Surgery, University of Milano-Bicocca, Milano, Italy; g Department of Haematology, Copenhagen University Hospital – Rigshospitalet, Copenhagen, Denmark

**Keywords:** Symptom science, phenomenology, Ricoeur, qualitative study, acute and chronic illness, symptom experiences

## Abstract

**Purpose:**

Exploring how patients with acute and/or chronic illnesses experience symptoms.

**Methods:**

Using a phenomenological approach, interviews were conducted with patients to gain insights into their lived experiences and the meanings they attribute to symptoms. Phenomenological concepts of lifeworld, body and embodiment, intentionality, and existential anxiety provide lenses for examining the realities of patients’ experiences.

**Results:**

Three central themes emerged from the Ricoeur inspired analysis: Perception of an altered body presence, which highlights how illness transforms the perception of the body from a “silent” part of the self to an intrusive presence; Symptoms as a threat to existence, illuminating how symptoms confront patients with vulnerability, mortality, and existential uncertainty; and Loss and reclaiming of control, describing the ongoing struggle patients face between feelings of helplessness and efforts to regain autonomy in daily life.Symptoms are not merely indicators of illness but are intertwined with patients’ identities and their sense of meaning in life.

**Conclusion:**

Insights emphasize the importance of addressing both the biological and existential dimensions of symptoms. Face-to-face clinical encounters offer a shared opportunity to create reflective spacesValidating coping strategies and supporting patients in reclaiming control, clinicians can nurture resilience, dignity, and a comprehensive approach to symptom (self)management.

## Introduction

In an era marked by increasing survival rates among patients grappling with acute and chronic illness, the resulting rise in illness- and treatment-related symptoms is a pressing concern. While existing research provides valuable insights from clinician-derived and patient-reported perspectives, the subjective experience of symptoms often remains underexplored. This study draws on symptom phenomenology, focusing on how symptoms arise, are experienced and impact various aspects of life. By exploring these lived experiences, clinicians and researchers can better understand and address the needs of individuals facing health challenges. This approach not only informs tailored interventions and treatment plans but also supports effective strategies for managing symptoms in a way that is meaningful to patients, ultimately enhancing patient outcomes and enriching the field of symptom science. As societies adapt to an increasing number of individuals living with chronic illness, understanding how people experience and manage symptoms in daily life is essential—not only to improve treatment outcomes, but to promote sustainable well-being and participation in society.

## Background

As survival rates increase in patients with acute and chronic illness, more patients are burdened by illness and/or treatment-related symptoms (Adam et al., 2023; Bennett et al., 2019; Cancelliere et al., 2016). Undetected and untreated symptoms are not only distressing but may disrupt optimal treatment, requiring changes to the treatment, complicate recovery, and delay hospital discharge. High symptom burden is associated with increased mental and physical impairment, greater health care consumption, and failure to return to work (Cai et al., 2024; Joo & Liu, 2022; Leger et al., 2015; Sheehan et al., 2019). Symptom control is among the most important patient needs across diagnostic groups and treatment contexts. Patients’ symptom experiences, self-reported symptom assessment and other targeted and sensitive biological, psychological, behavioural, and social outcomes are fundamental for assessing illness activity, recurrence as well as care and treatment effects (Dineen-Griffin et al., 2019; Joly et al., 2019). Thus, optimizing symptom management can significantly improve patients’ quality of life and well-being (Hassett et al., 2022). However, rooted, as we are, in healthcare within a contemporary biomedical model of disease (Carel, 2017; Degerman & Carel, 2025; I. J. Kidd & Carel, 2025, 2017; Toombs, 2001), it is tempting to direct most of our attention to a conceptual definition of symptoms from a clinician-derived perspective or as a patient-reported outcome that encompasses the number of symptoms, symptom severity, or the sum of symptom scores (Gill et al., 2012; Missel & Bergenholtz, 2020; Toombs, 2001). Such knowledge is highly needed and offers significant value in explaining and treating symptoms (Christiansen et al., 2023; Pappot & Taarnhøj, 2020; Tolstrup et al., 2019). While this approach provides important insights, it risks reducing health and illness to measurable and quantifiable components, which may overlook the subjective, existential, social, and cultural factors that contribute to an individual’s experience of symptoms (Benner, 2022; I. Kidd & Carel, 2025; Toombs, 2001). While quantitative data provide valuable insights, they primarily measure objective aspects and may fail to fully capture a patient’s lived experience. An understanding of symptoms’ meaning and impact from the patient’s perspective is thus essential for treatments to be both effective and meaningful. Unfortunately, current research insufficiently addresses this patient perceived aspect (Andersen et al., 2022).

The Precision Symptom Care Research Program (PROSPER) was initiated as a comprehensive multidisciplinary initiative that seeks to explain and understand complex symptoms in patients during and after specialized treatment at Copenhagen University Hospital, Rigshospitalet, Denmark. The programme aims to enhance symptom science and improve care across various illnesses and contexts. Guided by the National Institutes of Health Symptom Science Model (Cashion et al., 2017; Kurnat-Thoma et al., 2022) and the Symptom Management Model (Brant et al., 2010), PROSPER provides a clinical framework for assessing, measuring, and managing symptoms to meet the need for symptom control. As part of PROSPER, issues that will be explored in this study include how patients with acute and chronic illness perceive the notion of symptoms, and what influences their sense of symptoms during illness and treatment. In this study we define chronic illness as a long-term health condition that may not have a cure and can affect a person’s functioning and quality of life, such as cancer, neurological disorders, or autoimmune disease (World Healt Organization, 2024). The subjective nature of symptoms, patients’ feelings, thoughts, sense of control and circumstances, all play a role in how symptoms are understood and informs everyday symptom (self)management. This study aims to empirically explore symptom phenomenology in individuals living with illness, allowing them to describe their own experiences rather than using predetermined categories (Carel, 2011, 2017; Norlyk et al., 2023; Zahavi & Martiny, 2019). The purpose of this study is to explore the experiences, perceptions, and meanings patients with acute and/or chronic illnesses attribute to their symptoms. This exploration complements existing clinician-derived and patient-reported perspectives, offering insights into the human response to illness and informing personalized approaches to symptom (self)management and healthcare.

## Methods

This study is a qualitative investigation based on data from interviews with individuals in outpatient or inpatient care for chronic and/or acute malignant or non-malignant illness. In our exploration of the phenomenology of symptoms, we investigate how individuals subjectively experience and perceive their symptoms, aiming to illuminate the nuances and underlying structures that shape these experiences (Carel, 2011, 2017; Galagher Zahavi, 2012; Schiermer, 2013; Spence, 2017; Toombs, 2001).

### Philosophical underpinnings

In this study the phenomenology of symptoms encompasses an analysis and interpretation of narratives from individuals dealing with illness- and treatment-related symptoms. We are interested in their lived experiences of symptoms, which involves exploring how these individuals narrate and make sense of their symptoms in their own words. The phenomenological field of research is, however, not concerned with private thoughts but rather intersubjectively accessible modes of appearance (Gallagher & Brøsted Sørensen, 2006; Zahavi, 2003). In other words, this study is not focused on the patient’s experiences as merely their own private and subjective (psychological) experiences; rather, we are interested in structures only insofar as they represent common experiences (Gadamer, 2004; Ricoeur, 1973; Svenaeus, 2019). Such an analysis will reveal certain invariant (or typical) features, separate from the variations in their concrete manifestations (Toombs, 2001).

Accordingly, this study is grounded in a phenomenological research epistemology and methodology underpinned by Ricoeur’s narrative philosophy and interpretation theory. Inspired by Ricoeur, our epistemological stance for exploring first-person accounts of symptoms begins with his narrative philosophy in which narratives are recognized as a primary way for people to create coherence in their lives (Missel & Birkelund, 2019; Ricoeur, 1984b, 2002). The research process follows Ricoeur’s thoughts and moves from pre-reflective experiences (prefiguration) to narrative articulation (configuration), and finally to interpretation (refiguration), leading to a deeper understanding of symptoms beyond direct description (Missel & Birkelund, 2019). While phenomenology in this study serves as the lens through which we examine universal perspectives of symptom experiences, we follow Ricoeur by grafting a hermeneutic onto phenomenological philosophy. His point is that what is accessible to us in language requires interpretation to be understood more comprehensively (Missel & Birkelund, 2019; Ricoeur, 1973, 1976, 1998, 2002).

### Theoretical inspiration

While phenomenological methods were originally developed for philosophical inquiry, their concepts remain valuable for empirical research. Recognizing that there is no “view from nowhere”, we use phenomenology to “front-load” conceptions into our analysis (Gallagher & Brøsted Sørensen, 2006). In doing so, we apply phenomenology to qualitative data, enriching our understanding of the lived experiences of the participants. The following concepts—lifeworld, body and embodiment, intentionality, and existential anxiety—serve as critical lenses through which we examine the multifaceted reality of how symptoms are perceived, experienced, and managed in clinical contexts.

The *lifeworld* encompasses the everyday reality we often take for granted—our pre-scientific realm of experience that we are familiar with and typically do not question (Zahavi, 2018). The phenomenological dictum *“to the things themselves”* (Galagher Zahavi, 2012) can be interpreted as a call to return to this immediate experience. Our lives are not driven by theoretical considerations, but by practical concerns and our lived experiences. Understanding the lifeworld is significant in our empirical study of symptom phenomenology, as it allows us to appreciate how patients navigate their experiences of symptoms within everyday contexts. By examining symptoms through the lens of the lifeworld, we gain deeper insights into how these experiences are shaped.

From a phenomenological perspective, the *body* is central to how we experience the world. It is through our bodies that we interact with and perceive everything, including illness (Merleau-Ponty, 2004, 2009). The body is both a means of experiencing and an object we can observe, as Husserl (Galagher Zahavi, 2012) noted, distinguishing between the objective body (the body we have) and the lived body (the body as we experience it). *Embodiment* refers to how consciousness and experience are rooted in the body, shaping our identity and actions (Carel, 2011; Merleau-Ponty, 2009). This perspective helps us understand how patients experience and manage symptoms, as their bodily state impacts their lived experience.

In the phenomenological tradition, consciousness is always consciousness-of-something, a concept known as *intentionality* (Galagher Zahavi, 2012). Intentionality refers to how our consciousness is always oriented towards something—whether we are perceiving, judging, feeling, or thinking. This direction gives meaning to our perception, which is influenced by our emotions, moods, past experiences, and interests. These factors shape not only how the world appears to us but also how we understand it. Therefore, to understand what a symptom is from the patient’s perspective, we must examine what is intentionally represented.

Heidegger distinguishes *anxiety* from fear. Fear is directed at a specific, localizable object, while anxiety is not tied to anything particular; it concerns our relationship with the world itself and relates to an overproduction of meaning imposed on life (Heidegger, 1967). Anxiety is not a sudden panic but a subtle unease that disrupts our everyday life. It challenges our sense of familiarity, revealing how the roles we assume and the meanings, actions, and existential affiliations we find in things are revealed as contextual constructs (Heidegger, 1967). From a philosophical perspective, anxiety is a mood that shapes how we experience the world, with the meaning of things shifting according to our emotional state. Heidegger’s analysis of anxiety provides a framework for understanding the existential meaning of living with symptoms.

This study draws on excerpts from the *Handbook of phenomenology and medicine* (Toombs, 2001) illustrating the relevance of phenomenology in clinical medicine and healthcare. The texts clarify the distinction between immediate pre-theoretical experience and theoretical, scientific accounts. This distinction is important when investigating phenomena of symptoms from firsthand lived experiences. Understanding symptoms in their full complexity holds significant practical value in clinical contexts.

### Participants

Participants were purposefully selected from four populations: individuals receiving treatment for lung cancer in a thoracic surgical department, gynaecological or brain cancer in an oncology department, and myasthenia gravis in a neuromuscular outpatient department. These diverse illness and treatment contexts were chosen to explore symptoms from varied perspectives, focusing on *the person* rather than the medical condition. Recruitment was conducted by four of the authors (KP, LKA, MCM, and MM), who facilitated the selection of ten participants from various regions in Denmark. Participants were selected based on their willingness to participate and their ability to provide insight into their symptoms and experiences. All approached patients agreed to participate, and recruitment took place either during scheduled visits or through prior connections with their designated HCP. While no patients declined participation, data are unavailable from those who were not approached, were ineligible for inclusion, or chose not to participate. Interviews were conducted between March and July 2024, and participant characteristics are presented in Table I.

**Table I.** Participants.

Participants: Age, years median (range) Gender (female/male)	*n*=10 62 (41–78) 8/2
Family situation: Living with partner/family Living alone	7 3
Education: Primary school or less Further education Completed secondary school or higher	4 5 1
Job situation: Working full-time Working part-time Early retirement Retired due to age Sick Leave due to current illness	0 0 5 3 2
Diagnosis: Lung cancer Gynaecological cancer Brain cancer Myasthenia Gravis	4 2 1 3
Time since diagnose, years median (range)	3 (14 days-26 years)
Treatment received*: Surgery Medical treatment	5 9
Comorbidities: DM2 Hypertension Chronic obstructive pulmonary disease Neuropathy Back pain Metabolic disorders Gastroesophageal reflux disease	1 2 1 1 2 1 1

*The total number of treatments received does not equal the total number of participants, as some have undergone both surgical and medical treatments.

### Data collection

According to Ricoeur we experience the world in a pre-thinking way, however, such unarticulated experiences can be approached and illuminated through a process of narration (Missel & Birkelund, 2019; Ricoeur, 1984b, 1991). A narrative approach was therefore used for data collection during individual interviews through which the participants expressed their experiences as they saw them and wanted to present them. Openness in the interviews provided a starting point for allowing participants’ lived experiences to be voiced. Thus, the focus remained on being open to what participants had to say about the symptoms of a life with illness. Interview topics were guided by curiosity (Spence, 2017) regarding how the participants understood symptoms in their daily life with illness and the meaning they attached to them. As such, the interview process was open and unstructured, but aimed at illuminating experiences and perceptions of symptoms. Table II lists the interview questions; however, these questions were not strictly followed linearly but were used to facilitate dialogue. When recounting their stories, participants brought about a configuration themselves. All interviews were conducted face-to-face, either in person or via video call, by experienced qualitative researchers. The interviews lasted an average of 57 minutes (range: 30–75 minutes) and were recorded and transcribed verbatim. According to Ricoeur, when spoken words are transcribed, their meaning is freed from the original event and author’s intent (Missel & Birkelund, 2019; Ricoeur, 1998). Thus, the interpreter’s task is not to uncover the patient’s psychology but to interpret the issues the text reveals.

**Table II.** Interview guide.

Can you talk about how you experience living with your illness?
Can you talk about how you experience` your symptoms in daily life?
Can you describe how your symptoms have changed over time?

### Research team and reflexivity

The research team consisted of a multidisciplinary group including a neurologist, an oncologist, a physiotherapist researcher, two PhD nursing students, three postdoctoral nurse researchers, one clinical nurse specialist, and three senior nurse researchers/professor/associate professors. The team have substantial experience in phenomenological research and/or symptom science. Our diverse clinical and academic backgrounds informed both data collection and interpretation, with awareness of our own preunderstandings and theoretical commitments.

### Data analysis and interpretation

According to Ricoeur, humans leave traces in their utterances, which are often ambiguous and concealed (Missel & Birkelund, 2019; Ricoeur, 1973, 1984a, 1998). The goal of interpretation is not to recreate the “original”, but to unfold the reality and possibilities expressed in the text (Ricoeur, 1976, 1998). Therefore, the aim of interpretation is to reveal possible ways of understanding the traces left in the participants’ narratives about their symptom experiences. This process involves navigating layers of meaning, beginning with an initial search for overarching meaning, followed by structural analysis, and culminating in a comprehensive interpretation (Ricoeur, 1976). We started by repeatedly reading the narratives to form an initial “naïve” understanding. The structural analysis focused on the text’s internal composition, removing external context, leading to a more objective and explanatory understanding. This stage allowed us to move beyond surface-level understanding and interpret the deeper meaning, which is described by Ricoeur (1976) as *“understanding what is talked about”*. To reach this deeper level, we identified meaning units related to the experience of symptoms across interviews. These units were grouped based on experiential resemblance, and through interpretive reading, we abstracted subthemes and main themes. This process involved a dialectic movement between parts and whole, text and understanding, in accordance with phenomenological hermeneutic principles.

The process thus revealed patterns, subthemes, and main themes from the narratives, grounded in phenomenological concepts, providing insight into the participants’ lived experiences and forming the foundation for describing the study’s findings. In this study, themes refer to meaning structures derived through interpretive, phenomenological hermeneutic analysis, rather than thematic categories in a traditional coding sense. The comprehensive interpretation aimed to connect the text’s world with that of the researchers, transcending individual findings to explore broader, universal meanings. The structural analysis thus underwent further discussion, incorporating relevant theories and studies, as we delved deeper into the interpretation and discussion of the identified themes. Accordingly, this interpretive understanding provided deeper insight into the lifeworld phenomena of symptoms embedded in the participants’ narratives (Ricoeur, 1998). While our initial interpretation was inductively grounded in participants’ narratives, the subsequent comprehensive understanding draws on phenomenological theory not to apply it deductively, but to deepen the meaning of the phenomenon under study. This follows Ricoeur’s idea of distanciation and the hermeneutical spiral. The tension between lived experience and narrative expression is, however, well recognized in phenomenology. In particular, symptoms may obstruct not only bodily function but also the ability to articulate experience—introducing silences, distortions, or absences that are crucial to consider in phenomenological interpretation.

### Ethical considerations

The study was conducted in accordance with the Declaration of Helsinki (2013). According to Danish law, interview studies that do not involve biomedical interventions do not require approval by the regional Committees on Health Research Ethics. This study was therefore not submitted for ethical review by a health research ethics committee. However, the study was approved by the Danish Data Protection Agency (*p*-2023–15293) and adhered to the guidelines of the Danish National Committee on Health Research Ethics. Participants received both verbal and written information about the study’s purpose, the voluntary nature of participation, the right to withdraw at any time without consequences for their treatment, and measures to ensure confidentiality and data protection. Informed written and oral consent was obtained from all participants prior to participation. To ensure privacy and emotional safety, interviews were conducted in settings chosen by the participants. All interview data were anonymized using identification codes, and personal identifiers were removed. Data were securely stored and only accessible to the research team.

## Results

This study explores how patients experience symptoms while living with acute and/or chronic illness, revealing three central themes: *Perception of an altered body presence*, highlighting how illness transforms the perception of one’s body; *Symptoms as a threat to existence*, reflecting the existential challenges faced by patients as they confront their vulnerability; and *Loss and reclaiming of control*, illustrating the ongoing struggle between the desire for autonomy and the feelings of powerlessness. Together, these themes reveal how symptoms shape patients’ lived experiences.

### Perception of an altered body presence

This theme illuminates how patients perceive their bodies as both a source of symptoms and something foreign when illness strikes. The body, once a natural part of the “self”, becomes something that demands constant attention and control. Changes in bodily functions lead to a sense of alienation, manifesting both physically and emotionally. For instance, physical weakness, loss of former abilities, or chronic fatigue can create a persistent awareness of the body’s fragility and unpredictability. One patient describes the sensation of her arms *“dragging behind her”*, highlighting the profound experience of fatigue. Another patient describes how fatigue alters the body’s presence, causing it to react unpredictably.

…my neck feels heavy, and my shoulders slump… But it’s really hard to explain. Then I don’t get anything done, and I feel guilty, but if I push through, there are after-effects… I’ve tried, and then I get nauseous… That happens when I’ve overdone it. But it could also be a muscle issue; my illness affects my muscles …

Patients express a duality between the “I/self” and the body. A distinction central to phenomenology, where the body is often understood as the primary means through which we engage with the world. Symptoms disrupt this bodily existence, resulting in a struggle between the will to act and the body’s physical limitations. The body may be experienced as a constant presence, with symptoms that persist and never fully subside.

I can live with double vision. Not all the time, but sometimes. If it has to be… I’ve gotten used to it… and yet I haven’t. The constant reminder of my illness is tiring, but I try to encourage myself. Because it is annoying, and limiting, and not me… What I fear most is not being able to ride a bike anymore.

Symptoms interrupt the normal relationship between the body and the world, requiring conscious attention to navigate once automatic situations. This creates a sense of estrangement and requires planning. Thus, symptoms serve as a constant reminder of illness, even when they are absent or in remission. One patient describes the feeling of *“walking on paper”* and tingling in his fingers at night. These symptoms extend the illness’s presence in the body, requiring patients to adapt and renegotiate their sense of bodily presence. Even when not considered a major issue, symptoms remain part of daily life, creating a gap between intention and ability, leading to feelings of inadequacy and isolation. The body, once a tool for action, becomes a disrupted presence, hindering engagement. Shortness of breath is another pervasive symptom that limits activity and intensifies a sense of being trapped in one’s own body. The struggle to breathe, once an automatic and stable bodily function, transforms into a conscious battle, alienating the connection with the body.

My breathing has been the most challenging. I’ve gotten used to it, but it impacts my daily life. For example, when gardening, I can only do it for a short time before getting out of breath. It’s frustrating because it limits what I can do. Everything has to be adjusted to my pace, and my children even mention how they enjoy walking faster when I’m not with them…

Symptoms create a gap between intention and ability, leading to feelings of inadequacy and isolation. They alter the patient’s relationship with the body, turning it into an object of illness that impacts daily life and identity. Normally a “silent” presence in the lifeworld, the body demands attention when illness disrupts its functioning. Patients must plan activities around symptoms, constantly weighing what they can handle. The body becomes a source of uncertainty, challenging self-perception and the lifeworld. Through the body, the illness manifests, and patients interpret their situation based on this experience.

The symptoms come from time to time… I can tell when the cancer is back. When I’m treated, the cancer disappears, but then it comes back. And I can feel it, like… in my body… I can sense it. It’s like there’s a tightness. I don’t have pain, but I can just feel that it’s there.

Patients also reflect on how their symptoms can come and go, with their unpredictability creating a persistent sense of uncertainty. These shifting periods underscore the dynamic nature of the illness, where the body cannot be relied upon to behave consistently. Patients often feel trapped, lacking both control and insight, especially when they are unsure about the origin of their symptoms. One patient, for example, struggles to discern whether her symptoms are linked to COPD or lung cancer, which makes it difficult to make sense of what is happening with her body.

My breathing was different in November and December due to my COPD, but now it feels connected to the lung cancer … I can’t figure it out because it’s a completely different kind of difficulty. Everything overlaps, and I struggle to define what’s causing it. It feels like pressure on the lung, but it could also be my stress from the whole process …

Such confusion can exacerbate the feeling of loss of control and make it harder to find calmness or security. This can create a sense of disconnection from one’s body, which may appear as a mystery that cannot be solved. When the boundaries of symptoms blur this way, the altered presence of the body becomes a source of uncertainty, leading to questions about what it means to be ill and what the future holds. This can lead to a striving to understand and navigate an unfamiliar and uncertain reality.

### Symptoms as a threat to existence

In this theme, we explore how symptoms can trigger existential challenges, confronting patients with their vulnerability, mortality, and uncertainty about life and future. For some patients, regardless of diagnosis, illness brings symptoms of fear and existential anxiety. While anxiety may arise as a direct symptom of illness, other symptoms can also evoke existential anxiety, heightening an awareness of mortality. Several patients describe how death shifts from an abstract concept to a present reality. One patient reflects on this shift: *“I think it’s scary… they can’t remove it, but they can keep it under control, but I don’t know how much time I have left. I try to hide from it, even though it’s difficult, because… I feel like it’s so unfair”*. This anxiety reflects a shift in existential reality, where the body, once a source of stability, now represents existential uncertainty where even small bodily changes become interpreted as potential threats.

Anxiety is deeply intertwined with symptoms, shaping a daily life that feels unstable and unpredictable. This makes it difficult for patients to plan and engage in life as before. Symptoms are not just physical limitations but also an existential burden, forcing patients to confront their vulnerability in new ways. Anxiety stems not only from physical discomfort but also from the illness’s unpredictable and potentially life-threatening consequences. One patient illustrates this, emphasizing the close interplay between physical and psychological symptoms: “*It turns into anxiety. Not like I have an anxiety attack. I can feel that I overthink it… Why do I have this now? Should I Google it? I start thinking too much… so many of the symptoms I have, I convince myself it’s probably it [cancer]”*. This reflection highlights how physical symptoms can translate into existential concerns. The body is no longer viewed as a stable entity but as a source of threats, which further complicates the experience of symptoms turning them from simple signals of illness into existential markers. The fear of recurrence, or “flare-ups” in chronic illness, can add to this existential anxiety, compounding patients’ sense of uncertainty. This is evident when a patient describes the experience of suddenly being “caught” by physical limitations, such as being unable to walk against the wind due to leg fatigue: *“I become incredibly insecure about myself, and then I get really upset, it’s like a crisis … I don’t have the psyche for that…* Such moments encapsulate not only the physical burden but also the existential distress triggered by these symptoms, as they challenge the patients’ sense of self.

Patients’ experiences of symptoms often represent an existential dilemma raising questions about identity, control, and the experience of illness. These symptoms are not merely physical phenomena; they become markers of change, inviting reflection on the self and its place in the world. One patient describes how *“my eyes have become particularly sensitive to light”* and how this new symptom affects her interaction with the world around her. This heightened awareness of the body as a source of discomfort challenges the prior “silent” role of the body as a tool for navigating life, creating a sense of disconnection from the self. The body, once a reliable means of moving through life, becomes a constant reminder of vulnerability and limitation, raising existential questions about how to live with these changes and the uncertainty they bring. For others, symptoms intensify existential tension. A patient experiencing double vision and muscle weakness describes these limit not only physical abilities but the sense of agency and control.

It is the ruin of my life at the moment. It’s not control… I can’t give anything to anyone…, the feeling of not being able to give or to cope… But I think things affect each other, so if you get tired in your muscles, you might need to use more energy to think or be present. And that gives me a different perception of myself, which I’m not too fond of.

This is an example of how the physical symptoms disrupt not just the body’s functionality but also the patient’s sense of autonomy and self-worth, creating a dissonance between the way he once understood himself and the person he is becoming in response to illness. Thus, symptoms are not just physical experiences but are tied to existential reflections on identity, autonomy, and the body’s role in life.

Patients may find it difficult to distinguish between symptoms arising from different illnesses. This uncertainty about the origin of symptoms not only provokes anxiety but also contributes to the broader existential dilemma patients face; what does it mean to be ill? When symptoms become indistinguishable, the patient’s understanding of their body and illness is clouded, which deepens their existential questions.

The neurologist asks if I get fatigued…, and I think I do, but I’m also tired…, a kind of tiredness that is always there, but I also have an underactive thyroid. But now I’m on sick leave…, and it’s just so frustrating, because you don’t know where you stand. What does the future bring? And what can you do? Can we stay in the house? Can we … , all sorts of small and big things…?

This sense of uncertainty about the body’s condition is not only frustrating physically but also represents a threat to existence, fuelling existential concerns about identity, purpose, and the future.

### Loss and reclaiming of control

In the following, we explore how patients experience both the loss and reclaiming of control over their bodies and lives when faced with illness. Symptoms, as discussed in the previous themes, often embody a sense of unpredictability that challenges patients both physically—through experiences such as fluctuating fatigue or other bodily sensations—and existentially, by evoking anxiety related to illness progression or its impact on daily life. This interplay between losing and regaining control reflects the ongoing tension between vulnerability and resilience, where the process of reclaiming control may not always result in fully restoring control but represents an active striving to regain influence over one’s body and life. This effort occurs alongside the need to adapt to new limitations and cope with the loss of control in daily living.

Patients express a strong need for control to feel secure in their treatment and daily lives, striving to maintain agency despite bodily instability. One patient describes structuring her day to manage energy and symptoms.

I often make adjustments if I have something to do, so I can push myself a bit more… I get up, take my morning medication, then rest until lunch. If I have to go out, I know I can manage more if I’ve rested as much as possible. So, I’m constantly planning everything.

This planning reflects the tension between autonomy and symptom constraints. While careful structuring provides a sense of control, the unpredictability of symptoms underscores ongoing vulnerability. Patients thus seek normalcy within the limits imposed by their condition. Patients also describe cognitive symptoms that make daily activities overwhelming, even simple tasks like unloading a dishwasher: *“ … others can’t understand how hard it can be to empty a dishwasher”*. Cognitive exhaustion creates a sense of burden, complicating efforts to resume normal life and work. Tasks that were once automatic now require planning and effort. The same patient reflects on adjusting to “the new life”.

You spend time and energy figuring out your new life. It doesn’t just come back automatically … You rebuild it, stone by stone. Navigating constant fatigue, trouble concentrating, or pain means reconstructing everything – relationships, habits, work. It’s like everything is in play.

This example highlights the challenge to patients’ sense of control and agency. The perceived loss of control creates a gap between identity and lived reality, leading to frustration and diminished self-worth. One patient describes how she, once highly organized, now relies on lists and calendars: “*I’ve always had everything under control—planning with work, friends, and at home. But then I started forgetting … more and more slip-ups. It just got worse. I’ve never made lists before, but now I have to”*. This shift threatens her previous self-image as a competent and independent person, reinforcing the feeling of losing control over her body and life.

On the other hand, patients employ various strategies to regain control, ranging from openness about their illness, which invites connection and understanding, to emotional distancing or physical activity, which provides a buffer against overwhelming feelings. Patients often seek to restore a sense of freedom and autonomy by making active choices, even though these choices are limited by symptoms. The body’s resilience becomes a key resource in coping with symptoms.

As a lung cancer patient, you quickly become a living dead … People avoid you. I didn’t want that, so I told everyone from the start. Maybe I’ve been more open than most men, but it has strengthened my relationships – not just with my wife and children, but beyond them too. That openness keeps me from becoming one of the living dead.

This illustrates how openness can counteract stigma and foster connection, reinforcing a sense of control and agency. By sharing their experiences, patients “steer” the narrative of their illness, strengthening autonomy. They balance distance and closeness to symptoms—distancing helps maintain control, while acknowledging symptoms affirms their vulnerability. Avoiding confrontation can also preserve normalcy amidst the chaos of illness, acting as a form of existential shielding: “*When it comes to my symptoms … I think I shut down a bit. I’d rather not engage too much. I acknowledge I’m struggling to breathe, but I just tell myself, let’s see how things are tomorrow…”* Through such strategies, patients navigate the tension between vulnerability and resilience, coping with their symptoms while understanding their existence amidst life’s uncertainties. Distancing from emotional and existential burdens allows focus on the tangible aspects of symptoms to preserve a sense of control. One patient sees psychological states, like anxiety and depression, as *“consequences”* of illness rather than symptoms, reflecting a separation between body and mind. Another patient views anxiety during the wait for scan results not as a symptom, but rather as a natural part of being a *“thinking human being”*. This distinction helps patients manage psychological aspects as external to the disease, maintaining some control over their illness.

A significant aspect of patients’ experiences is the unpredictability of symptoms. Even those who have lived with the illness for years may struggle to identify triggers, creating a sense of instability and loss of control. This uncertainty challenges their sense of stability and freedom in daily life. Patients resist “capitulating” to their bodies’ limitations and constantly renegotiate control to find meaning in an otherwise uncontrollable situation. They actively manage their symptoms to preserve autonomy and dignity, showing resistance to passivity despite physical limitations: “*I have done a lot myself… For me, it’s better to confront it, name it, and figure out what to do about it. I can’t sit passively”.*

## Comprehensive understanding and discussion

The findings of our study highlight the phenomenology of symptoms in both acute and chronic illness, showing that symptoms are not just physical but also existential experiences that impact identity, agency, and the ways in which patients relate to their lifeworld. In this section, we will employ selected theoretical perspectives to interpret the findings to achieve a more comprehensive understanding (Missel & Birkelund, 2019; Ricoeur, 1976). The intention is to inform more personalized and “effective” approaches to symptom (self-)management in clinical practice.

Our findings illuminate how symptoms may challenge patients’ relationship with their bodies by bringing the body to the forefront as a conscious presence. From a phenomenological perspective, the body is typically experienced as an integrated and “tacit” part of the self, enabling seamless interaction with the world (Merleau-Ponty, 2004). However, when bodily capacities are disrupted, their significance extends far beyond mere mechanical dysfunction, as the relationship with the body is inherently existential (Heidegger, 1967; Toombs, 2001). When symptoms arise, this natural integration is disrupted, and the body becomes an object that demands attention and control. This not only alters patients’ relationship with their bodies but also their perception of themselves as acting subjects (Carel, 2011; Galagher Zahavi, 2012). This disruption can be understood in the context of the mind/body duality, where symptoms disturb the interplay and challenge the integration between mental and physical experiences. In this context, the concept of interoception (Locatelli et al., 2024) may offer additional insights. Interoceptive accuracy, or the ability to perceive internal bodily signals, has been found to be lower in patients with chronic conditions (Locatelli, Matus, et al., 2023). Moreover, interoceptive sensibility, which refers to the subjective tendency to notice and value these signals, appears to be associated with lower symptom severity and frequency. This underscores the complexity of how patients experience and interpret their symptoms. Complementary, from a phenomenological perspective, symptoms also introduce challenges in perceiving and interpreting bodily sensations, further complicating the mind-body interplay. The body is perceived both as a limitation and as an external entity that no longer aligns with the patient’s intentions and desires. This means that whatever happens to my body also happens to *me* (Merleau-Ponty, 2009; Toombs, 2001). Similarly, Missel et al. (2021) illuminate how patients’ existential anxiety and loss of”homeliness” in palliative care, render the body unhomelike, disrupting their sense of self‑world continuity. Such experiences raise fundamental questions about identity, as the patient is forced to relate to the body as both “self” and “other” challenging the fundamental sense of homelikeness within the body (Heidegger & Boss, 2001). For clinicians, this un-homelikeness highlights the importance of recognizing how symptoms fundamentally alter patients’ relationship with their sense of self. Furthermore, symptoms, through this duality, can alter the patient’s intentionality—the ability to direct oneself towards and act in daily life. Symptoms draw attention inward (Carel, 2017; Toombs, 2001), towards the body itself, creating a distortion of the lifeworld, where everyday tasks and future goals become secondary to managing the body’s demands.

An existential tension may be experienced, where the self appears fragmented between the acting intention and the body’s limitations. This is referred to as existential anatomy (Merleau-Ponty, 2009; Toombs, 2001; van den Berg, 1983), which describes how intentionality shifts from engaging with life itself to focusing on the contents of life. As a result, patients may experience loss of meaning in their lives, as their focus shifts predominantly to the body’s unfamiliar and unsettling signals, leaving less space for engagement in other meaningful aspects of life as also reported previously (Piil et al., 2022). Some patients may respond to this loss of control by either amplifying or minimizing their symptom experiences. Over-reporting of symptoms may reflect an attempt to express struggles, seek validation, or regain agency in the face of the body’s unpredictability (Kornelsen et al., 2016). Conversely, under-reporting may serve as a strategy to preserve a sense of strength or control, as patients attempt to maintain their routines and minimize the perceived impact of their symptoms. However, both approaches can complicate the clinical dialogue. In clinical practice, it is thus crucial to understand how this disruption in embodiment can affect the patient’s ability to accurately narrate about symptoms. Patients may also struggle to articulate certain bodily sensations that lie between existing semantic markers, making it harder to translate their lived experiences into words (Toombs, 2001). The experience of illness may also take on the character of a “saturated phenomenon”, as Grīnfelde (2019) draws on Jean-Luc Marion to show—a body that overflows experiential and affective capacity, disrupting habitual embodiment and resisting full conceptualization. This deepens our interpretation of symptom-induced un-homelikeness, highlighting how patients may feel overwhelmed by bodily presence that defies narrative integration. This struggle to articulate symptoms highlights the significance of the clinical encounter as more than a mere exchange of information. Recent empirical evidence supports this and underscore how existential disruption may manifest as narrative misalignment and symptom confusion (Li et al., 2024). In the clinical dialogue, patients lay open their world, facing the mirror of alterity. The clinical space becomes a relational space, where the patients address themselves to another, the clinician, and, through this encounter, gains an increased awareness of their own condition. This face-to-face dialogue is foundational for creating meaning and value, as patients reveal themselves through and with the help of the clinician (Lévinas, 2020). Recognizing the relational nature of the clinical encounter enables clinicians to foster a space where patients can explore and express their experiences. For example, recent observational work in diabetes and nephrology clinics demonstrates that trustful patient—clinician relationships facilitate negotiation between professional perspectives and patients’ lived experiences and thereby creating room for dialogue that carries meaning beyond mere information exchange (Christensen et al., 2024). Similarly, studies in primary care underscore how coordinated empathy and relationship with the patient contribute to emotional attunement, shared understanding, and therapeutic collaboration (Muramatsu et al., 2025). By engaging in this reflective dialogue, clinicians not only help patients navigate the interplay between mind and body but also support the restoration of a sense of meaning, autonomy, and self-understanding.

Symptoms, as described in our study, bring patients into contact with fundamental existential questions about their vulnerability, mortality, and future. Symptoms can transform daily life and the future from an open horizon into a source of uncertainty and anxiety, with the intentionality of the experience directing attention inward towards the presentation of a primary bodily symptom (Toombs, 2001). For instance, symptoms such as pain, fatigue, or shortness of breath may force patients to focus on immediate existential needs, limiting their ability to plan and envision the future. These changes to the lifeworld create existential strain, challenging patients’ understanding of themselves as active and future-oriented beings (Heidegger & Boss, 2001; Missel et al., 2021). This estrangement and loosening of the patient’s connection to the world are closely tied to biology (Toombs, 2001). Such inwardness is characteristic of many presentations of symptoms of predominantly organic disease, especially those unfamiliar to the patient. The meanings of these symptoms are rooted in the body-as-nature, which lacks the familiar logic or normality of existential anatomy (Toombs, 2001; van den Berg, 1983). As a result, it becomes difficult for patients to name and describe their experiences, and what the patient will tell can often be an abstraction from the actual lived experience. There is a gap between the experience as it is lived and the experience as it is narrated, leaving symptoms abstract until they are integrated into the realm of individual life (Frank, 2001, 2020). Therefore, in clinical practice, creating space for patients to articulate their symptoms is crucial, as it helps bridge this gap.

The signals of meaninglessness and threat may extend beyond the individual’s immediate awareness, highlighting that the patient’s body becomes a personal reality they are forced into, with limited control. This reality is revealed in moments of bodily change (Toombs, 2001). This disruption affects patients’ agency, as symptoms diminish their sense of control over both their bodies and their lives. Instead of acting freely and purposefully, patients are often forced to adapt to their symptoms, which can lead to feelings of helplessness, as unfolded in our analysis. This may be further compounded by the experience of symptoms as unpredictable or lacking clear solutions, undermining patients’ ability to navigate their situation with meaning and direction. Additionally, a recent study describes patients’ feelings of “powerlessness in the face of illness” when symptoms emerge unpredictably and care systems lack kindness or flexibility—leading to insecurity, frustration, and compromised agency (Walløe et al., 2024). In a clinical context, this means that clinicians should acknowledge and support patients in regaining agency and meaning. By understanding symptoms as more than just physical phenomena—but also as existential markers—clinicians can help patients rebuild a sense of control and direction in life. For example, this could be achieved through support that encourages patients to explore their goals despite the limitations imposed by their symptoms, which can enhance symptom self-management strategies (Christiansen et al., 2021). Additionally, the biological dimension is also important in a direct way in the understanding of symptom presentations and supporting patients (Toombs, 2001). Biomedical knowledge not only serves as a tool in its formal sense, but it also helps to meet the patient’s feeling of desolation within a discomforting body. When one understands the pathophysiological changes, it becomes easier to understand also the experiences caused by these changes.

Moreover, clinicians should be attentive to how symptoms may influence patients’ narratives about their condition. Understanding patients’ existential experiences can therefore contribute to a more accurate and meaningful dialogue about symptoms in clinical practice. The patient’s language of symptom presentation may, however, be heterogeneous and a mixture of spoken and body language, and the patient’s story is often fluid, unpredictable, and fecund, rather than emerging as a single, coherent account (Toombs, 2001). The face-to-face clinical encounter between patient and clinician holds particular ethical significance (Lévinas, 2020) serving as a starting point for the creation of meaning and value. In this clinical dialogue, the patient can reveal their “self”, calling on clinicians to witness, confront, and be responsive (and responsible) to the patient’s symptom experiences.

Patients’ efforts to regain control over their lives and bodies are a central aspect of how they navigate their experiences of symptoms. Despite the often intrusive nature of symptoms, patients in our study strive to maintain a sense of autonomy and dignity, which is expressed through various strategies that help them adapt to their symptoms and reduce feelings of helplessness. These strategies not only reflect patients’ resilience but also reveal their ongoing negotiation with the challenges posed by their bodies. One important strategy is emotional distancing, where patients create a deliberate separation between themselves and their symptoms. From a phenomenological perspective, this can be understood as a form of existential shielding, a concept that echoes Carel’s (2017) insights into how individuals living with illness develop adaptive mechanisms to preserve their lifeworld. By consciously detaching from their symptoms, patients can temporarily shield themselves, allowing them to function more effectively. Distancing also enables patients to preserve their identity as more than just “ill”, helping them continue to engage in meaningful activities that affirm their sense of self beyond the limitations imposed by their symptoms (Toombs, 2001).

Another common approach is structuring daily life, where patients plan and prioritize their time and energy to manage symptoms effectively. This represents a practical form of agency in the context of embodiment, as patients adapt their daily routines to the limitations of their bodies (Heidegger, 1967; Toombs, 2001). Such practical engagement with the world and the body is central to human existence (Heidegger, 1967) and can be understood as a way of renegotiating the relationship between intention and action, allowing patients to develop a new understanding of what they can achieve within the constraints of their symptoms. Coping through openness is also a strategy where patients choose to share their experiences with symptoms. By articulating their experiences, patients establish a framework for understanding and support from their social and professional networks, and thereby enhancing a sense of control (Frank, 2012, 2020; Toombs, 2001). Drawing on Frank’s concept of “telling-illness”, this process allows patients to experience their illness and symptoms as a story in which they are both narrator and the narrated, helping to organize their consciousness while also being shaped by it (Toombs, 2001). This narrative process enables patients to navigate their symptoms with a sense of connection and shared understanding, emphasizing that illness is not only an individual experience but also a relational one (Carel, 2017; Frank, 1995). In this way, openness allows patients to position themselves as active participants in their care and life, reinforcing their sense of identity and agency.

These strategies collectively reflect a profound human striving for autonomy and dignity in the face of the limitations imposed by symptoms. In this context, autonomy refers to the ability to make meaningful choices and maintain a sense of control over one’s life. Dignity, on the other hand, is manifested in patients’ efforts to preserve a positive self-perception and prevent symptoms from entirely defining their identity (Øhrstrøm & Dalgaard Mh, 2011; Vehling & Mehnert, 2014). Missel and Bergenholtz (2020) further emphasize this in the context of incurable oesophageal cancer, where maintaining lifeworld-centred care—through reverential response from clinicians and support for daily routines—was crucial to preserve patients’ dignity and sense of self. By employing strategies such as emotional distancing, structuring daily life, and openness, patients demonstrate a resilient engagement with their circumstances, actively working to balance the challenges of symptoms with their desire for a meaningful existence. In a clinical context, these strategies carry significant implications for practice. Clinicians can support patients by recognizing their individual coping methods and help them navigate symptoms effectively. This includes offering guidance on energy management, providing psychosocial support, and fostering open dialogue about symptoms and their impact on patients’ lives (Locatelli, Pasta, et al., 2023). By validating strategies such as existential shielding, clinicians can reduce feelings of isolation and strengthen patients’ sense of control. Encouraging patients to articulate their experiences in ways that integrate symptoms into their broader life story further enhances their agency and fosters a relational process that strengthens the therapeutic alliance between patients and clinicians (Damsgaard et al., 2021). Face-to-face dialogue should not be considered merely a threshold to action; it is the action itself. In dialogue, the patient not only reveals themselves outwardly but also becomes who they are; not only for the clinician but for themselves as well (Toombs, 2001). Together, these approaches promote a personalized and dignified approach to symptom management.

## Methodological considerations

In this study, we applied phenomenology to enhance patient care by gaining an understanding of patients’ lived experiences (Missel & Birkelund, 2019). Despite some debate over its appropriateness in clinical health research (Paley, 2005, 2017), we used phenomenology as a philosophical stance, known as applied phenomenology (Gallagher & Brøsted Sørensen, 2006; Zahavi & Martiny, 2019). This approach allowed us to explore the existential dimensions of illness, beyond clinical symptoms, offering insights into how patients perceive their bodies, symptoms, and identity (Galagher Zahavi, 2012; Schiermer, 2013; Svenaeus, 2019; Van Manen, 2017). A fundamental challenge in phenomenological research is the lack of direct access to first-person experiences (Svenaeus, 2019). Instead, we relied on patient narratives, which represent their attempts to make sense of their lived experiences (Ricoeur, 1984b, 1991, 2002). These narratives, though filtered through the patient’s interpretation and language, offer a valuable window into how individuals experience their symptoms. However, they cannot fully capture the immediacy or totality of lived experience, as meaning is shaped by both personal insight and the ability to articulate it (Ricoeur, 1984a; Ricœur et al., 2007). This highlights the limitations of interview-based data (Nunkoosing, 2005).

In phenomenological health research, concepts like validity, trustworthiness, and transferability are complex (Creswell, 2013; Morse, 2015b). In this study, we aimed to uncover shared meanings behind individuals’ experiences of symptoms, focusing on universal features of human existence. The validity of phenomenology lies in its ability to reveal these common features, distinct from individual variations (Galagher Zahavi, 2012; Spence, 2017). To ensure validity, we carefully interpreted patients’ descriptions to identify universal structures, guided by Ricoeur’s theory of narrative and interpretation (Missel & Birkelund, 2019; Ricoeur, 1976, 1984b, 1991). Regarding transferability, phenomenological research does not seek broad generalizability (Creswell, 2013). Instead, it aims to offer rich, detailed accounts of shared experiences that may resonate with others in similar contexts. By including a diverse sample, we explored symptoms across various illnesses, identifying common features that extend beyond specific diagnoses, offering a broader understanding of symptom phenomenology (Carminati, 2018; Creswell, 2013; Morse, 2015b). Although the sample size is small, it allowed for a focused exploration of shared experiences. We acknowledge potential “selection bias” due to purposeful sampling, but by focusing on universal aspects, we aimed to provide insights that resonate with individuals in similar contexts (Hennink et al., 2017; Malterud et al., 2016; Morse, 2015a). There are, however, also certain challenges associated with selecting diverse populations. Different illnesses and treatment pathways can lead to markedly distinct experiences of symptoms, affecting patients’ lives in various ways. This variation carries an inherent risk that individual patient experiences may be dominated by their specific situation, potentially overshadowing the universal aspects of symptoms we wish to highlight. However, our aim is not to erase individual variation, but to understand symptoms as phenomena that contain existential dimensions shared across illness trajectories. While phenomenological analysis seeks typical structures of experience, these insights can inform personalized care by sensitizing clinicians to underlying patterns of meaning that shape how patients live with and respond to symptoms. Thus, general understanding may serve as a resource for individualized care, rather than a contradiction of it. Therefore, throughout the research process, we have focused on common experiences, avoiding an overemphasis on the unique aspects of each illness. The sample had limited age variation, and only two males were included, which should be considered when interpreting the findings.

## Concluding remarks

This study highlights how symptoms profoundly shape patients’ lived experiences, altering their relationship with their bodies, their sense of self, and their engagement with the world. Symptoms pose existential challenges, confronting patients with vulnerability, mortality, and the struggle to regain control. Despite these challenges, patients demonstrate resilience through strategies that help them maintain autonomy and dignity. By recognizing symptoms as more than physical phenomena, clinicians can provide treatment and care that addresses not only the physical but also the existential realities of patients’ experiences. This underscores the need for personalized, empathetic, and ethically attuned approaches to symptom management in clinical practice.

## Relevance to clinical practice

Understanding how symptoms disrupt the body’s normal function is crucial in clinical practice, as it affects patients’ ability to communicate their experiences and maintain a sense of agency. Clinicians can help by creating spaces for reflection, allowing them to make sense of their symptoms. This involves empathetic communication that addresses both the physical and existential aspects of illness, helping patients reconnect with their values and goals despite limitations. The clinical relationship should also encourage critical thinking, challenging assumptions, and helping patients reconsider their perspectives. Practical approaches include guidance on energy management, emotional support, and addressing tendencies to under- or overreport symptoms. Recognizing these patterns as adaptive strategies allows clinicians to engage in communication that is responsive to the patient’s needs. By acknowledging and supporting patients’ active efforts to navigate their symptoms, clinicians can promote a personalized and dignified approach to care. Ultimately, this approach strengthens the therapeutic relationship, empowers patients to regain a sense of control, and fosters resilience in the face of illness.

## Data Availability

The first author/corresponding author (MM) and the last author (KP) have full control of all primary raw data (interview transcripts) and allow the journal to review our data if requested. The data generated during and/or analysed during the current study are available from the first author on reasonable request. All raw data are written in Danish. Data are stored in a locked file cabinet in a locked room at the Copenhagen University Hospital as requested by the Danish Data Protection Agency.
